# Disinfection of Scarlet Fever by Antiseptic Inunction

**Published:** 1893-07-15

**Authors:** C. T. Kingzett

**Affiliations:** Sanitas Company (Limited)


### EDITOR'S LETTER-BOX.

[Our correspondents are reminded that prolixity is a great bar to publication, and that brevity of style and conciseness of statement greatly facilitate early insertion.]

### DISINFECTION OF SCARLET FEVER BY ANTISEPTIC INUNCTION.

Mr. C. T. Kingzett, F.I.C., F.C.S., writes from the Sanitas Company (Limited) as follows: My attention has been directed to a letter by Dr. J. B. Curgenven in your last issue, having reference to this subject, wherein he advises the use of two particular varieties of eucalyptus oil, and adds that there is no necessity for the use of "Sanitas," as one of the eucalyptus oils named by him is a much more powerful antiseptic. I must, of course, assume that his remarks have reference to the particular "Sanitas" preparation known as "Sanitas" oil, as that is the only one which is qualified and recommended for use in such treatment, and that being so I respectfully beg to challenge Dr. Curgenven to adduce any chemical or other scientific evidence which will justify his statement. Most certainly on the other hand I assert that "Sanitas" oil is a far more powerful antiseptic, disinfectant, and oxidising agent than any variety of eucalyptus oil, and I challenge him to disprove this statement by any experimental evidence that would be acceptable to a body of scientific men qualified to judge of the matter. If Dr. Curgenven will accept this challenge doubtless means can easily be found for having a proper examination made on a basis satisfactory to both parties. In the meantime I may be allowed to protest in the strongest manner against such a totally unwarranted and hypothetical statement, which I am justified in stating from my own experience is absolutely opposed to the facts.

